# Supplementary material for: Spatial–Temporal Variations in Atmospheric Factors Contribute to SARS-CoV-2 Outbreak
Source: Viruses. 2020 May 27;12(6):588. doi: 10.3390/v12060588 (PMC7354543; doi:10.3390/v12060588)
Supplement: Supplementary file 1 [file viruses-12-00588-s001.pdf]

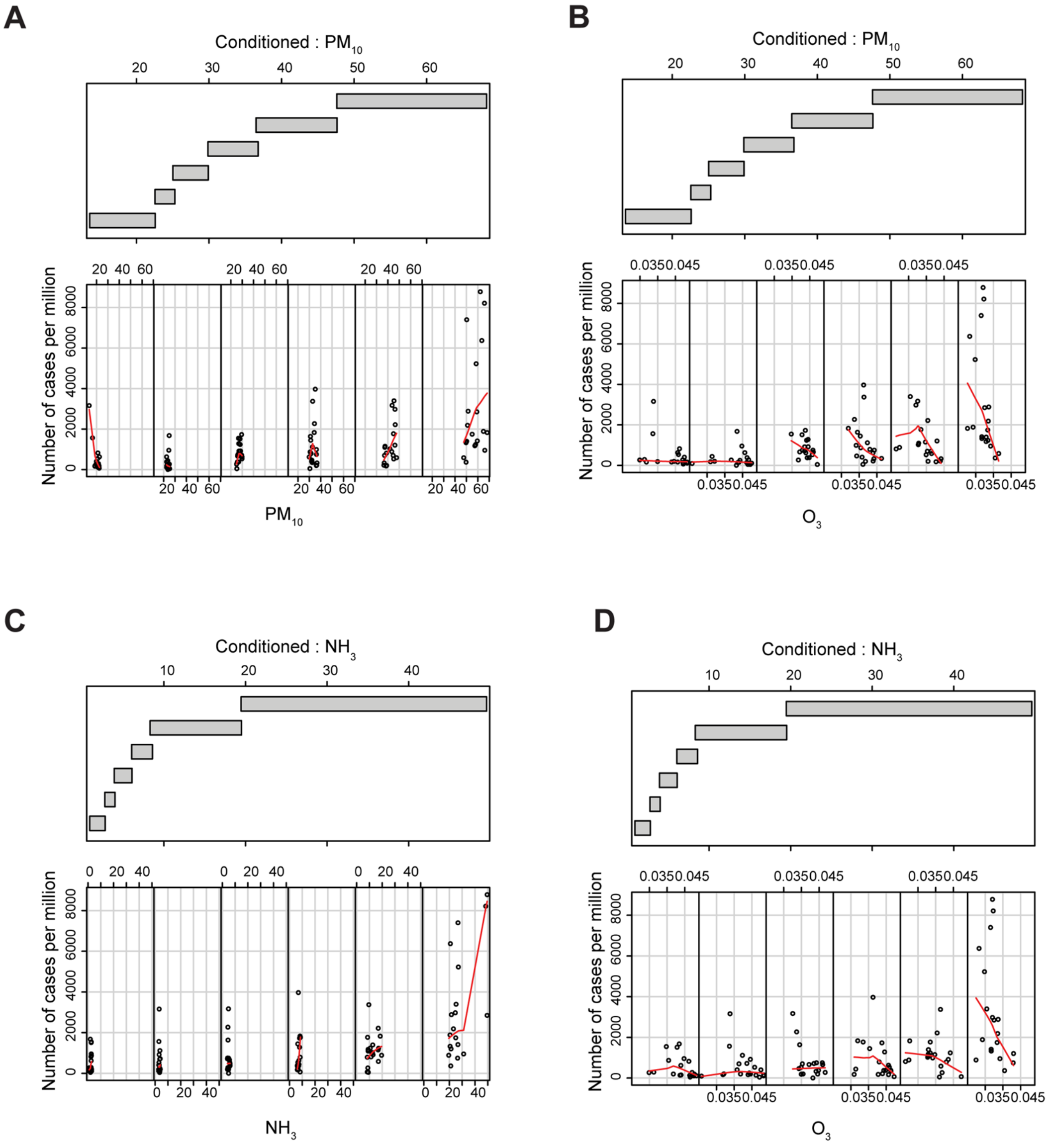

Figure S1. Evaluation of the cross-influence among PM_{10}, NH_3 and O_3. Conditioned plots with six levels of conditioning. Lower panel: the scatter plots between the selected factor and the number of cases per million. Each plot is restricted to show data points that belong to the provinces that fall in the corresponding range of the conditioning factor. Upper plot: the range of the values that define each level of conditioning. The overlapping among the levels is 0.1. The LOESS curve is computed in each scatterplot and shown in red. A) the scatterplot of PM_{10} conditioned to PM_{10}; B) the scatterplot of O_3 conditioned to PM_{10}; C) the scatterplot of NH_3 conditioned to NH_3; D) the scatterplot of O_3 conditioned to NH_3.

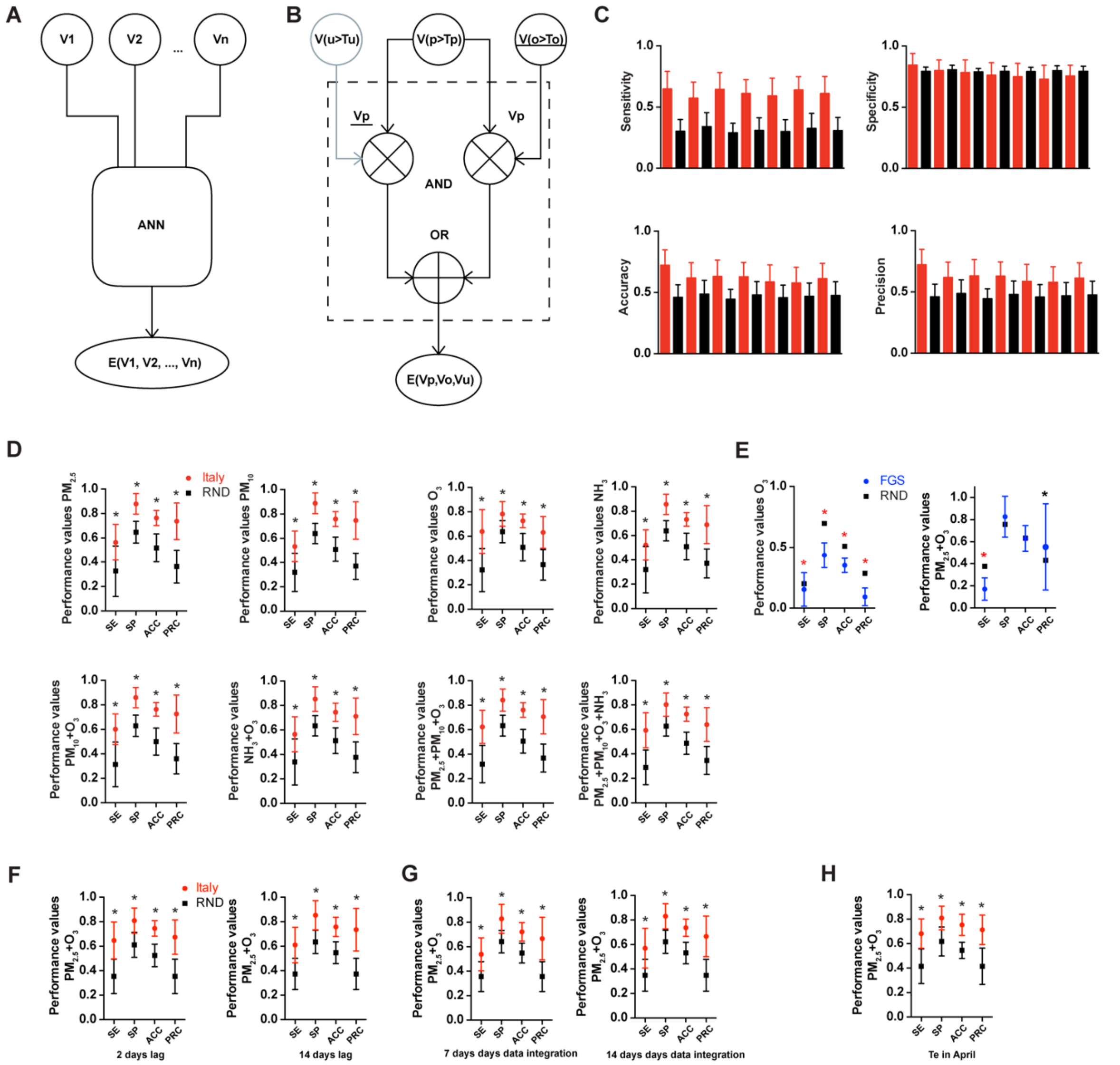

Figure S2. ANN and approximated logic model schema. A) The schematic design of the AAN classifier. A set of N input nodes are filled with a combination of the measurement of atmospheric factors V_1, V_2, …, V_n. The output of the ANN is a function $E(V_1, V_2, \ldots, V_n)$ on the atmospheric variables V_1, V_2, …, V_n. B) Intuitive boolean diagram of the model suggested by our conclusions. V_p, V_o, and V_u are three input signals that correspond to $PM_{2.5}$, O_3, and an unknown generic variable. The three variables are mapped to {0,1} using a threshold function where Vx=1 if the concentration of x is bigger than a threshold T_x and Vx=0 otherwise. The threshold T_x is implicitly determined by the neural network. The notation $\underline{V}$ indicates the negation of V. $\oplus$ represents an OR and $\otimes$ an AND gate. E(Vp,Vo, Vu) is the binary output of our model given V_p, V_o, and V_u. ANN performance evaluation. C) Performance indexes of the ANN varying the number of hidden nodes from 3 to 15 (step of 2). The bars represent the standard deviations of 100 Monte Carlo cross-validations. Italian provinces data are represented in red histograms, random data in black histograms. D) Performance values of the ANN on the 107 Italian provinces data varying the input variables. SE sensitivity, SP specificity, ACC accuracy, PRC precision. The dots represent the ANN average performance based on 100 Monte Carlo cross-validations. Bars represent the standard deviation. Italian provinces (red dots); France, Germany and Spain (FGS) regions (blue dots) and random dataset (black dots). E) Performance values of the ANN on the 47 FGS regions using atmospheric values ($PM_{2.5}$, O_3). FGS (blue dots), and random dataset (black dots). Performance values of the ANN on the 107 Italian

provinces data varying the input variables. SE sensitivity, SP specificity, ACC accuracy, PRC precision: F) lag of 2 and 14 days G) 7 and 14 days of average exposure H) Escalation threshold analysis on the 15th of April. Statistical analysis were performed using multiple t test corrected with Sidak-Boneferroni method for multiple comparisons (p <0.001). Red asterisk indicates that the classifier performs worse than the null classifier.

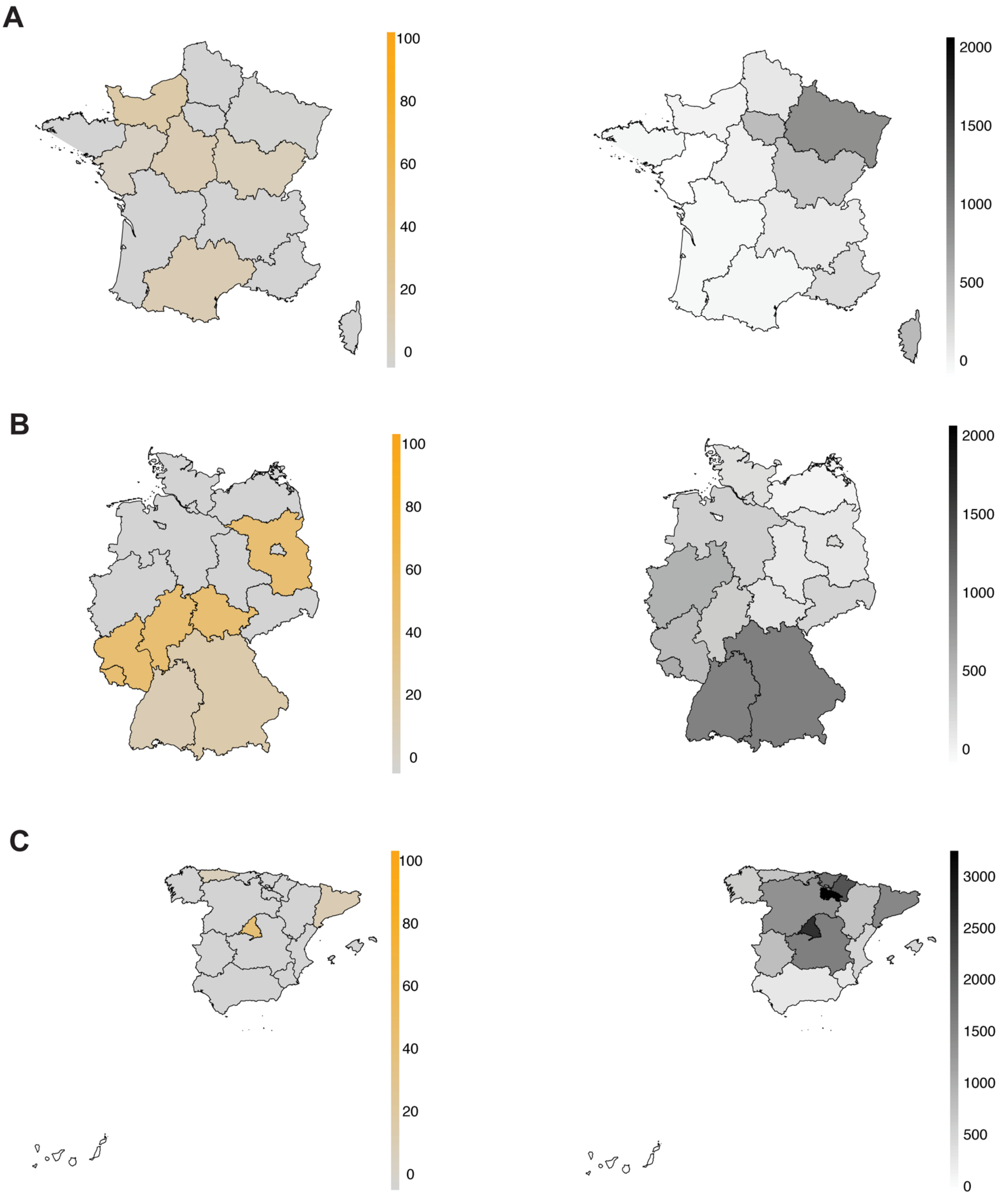

Figure S3. The spatial administrative maps representing prediction (orange) and actual reported cases per million (black) for A) France, B) Germany and C) Spain. Germany and Spain number of cases were limited to 2000 and 3000 cases per million to improve the dynamic range of the maps. The color intensity on the prediction maps represents the number of times that regions were positive for the outbreak in one hundred predictions.

Supplementary Table S1. Spearman coefficients.

	PD	PM_{10}max	PM_{10}sum	$PM_{2.5}$max	$PM_{2.5}$sum	NH_3sum	NH_3max	O_3max	O_3sum	TD	T	RH	WIND	P	Cas
PD	1.000	0.377	0.325	0.354	0.303	0.048	0.084	-0.244	-0.276	0.045	0.094	-0.108	0.006	0.188	0.034
PM_{10}max	0.377	1.000	0.989	0.990	0.985	0.762	0.787	-0.587	-0.792	-0.209	-0.136	-0.282	-0.441	0.269	0.586
PM_{10}sum	0.325	0.989	1.000	0.973	0.983	0.794	0.816	-0.561	-0.773	-0.142	-0.077	-0.234	-0.392	0.332	0.583
PM2.5max	0.354	0.990	0.973	1.000	0.993	0.765	0.787	-0.621	-0.820	-0.300	-0.221	-0.336	-0.520	0.192	0.597
PM2.5sum	0.303	0.985	0.983	0.993	1.000	0.796	0.815	-0.612	-0.818	-0.282	-0.209	-0.315	-0.511	0.213	0.598
NH_3sum	0.048	0.762	0.794	0.765	0.796	1.000	0.995	-0.468	-0.624	-0.237	-0.181	-0.251	-0.383	0.138	0.676
NH_3max	0.084	0.787	0.816	0.787	0.815	0.995	1.000	-0.472	-0.634	-0.227	-0.168	-0.253	-0.377	0.164	0.693
O_3max	-0.244	-0.587	-0.561	-0.621	-0.612	-0.468	-0.472	1.000	0.906	0.471	0.351	0.502	0.439	0.025	-0.444
O_3sum	-0.276	-0.792	-0.773	-0.820	-0.818	-0.624	-0.634	0.906	1.000	0.453	0.341	0.478	0.535	-0.052	-0.496
TD	0.045	-0.209	-0.142	-0.300	-0.282	-0.237	-0.227	0.471	0.453	1.000	0.942	0.566	0.792	0.789	-0.327
T	0.094	-0.136	-0.077	-0.221	-0.209	-0.181	-0.168	0.351	0.341	0.942	1.000	0.255	0.755	0.860	-0.284
RH	-0.108	-0.282	-0.234	-0.336	-0.315	-0.251	-0.253	0.502	0.478	0.566	0.255	1.000	0.432	0.155	-0.253
WIND	0.006	-0.441	-0.392	-0.520	-0.511	-0.383	-0.377	0.439	0.535	0.792	0.755	0.432	1.000	0.509	-0.327
P	0.188	0.269	0.332	0.192	0.213	0.138	0.164	0.025	-0.052	0.789	0.860	0.155	0.509	1.000	-0.025
Cas	0.034	0.586	0.583	0.597	0.598	0.676	0.693	-0.444	-0.496	-0.327	-0.284	-0.253	-0.327	-0.025	1.000

Supplementary Table S2. ANN performance.

Days	SE avg	SP avg	ACC avg	PRE avg	SE sd	SP sd	ACC sd	SP sd
27	0.644	0.862	0.780	0.737	0.140	0.092	0.064	0.136
14	0.570	0.831	0.737	0.666	0.162	0.103	0.070	0.166
7	0.538	0.828	0.721	0.666	0.135	0.118	0.076	0.174
27 RND	0.309	0.636	0.503	0.361	0.156	0.089	0.107	0.113
14 RND	0.349	0.622	0.531	0.349	0.130	0.094	0.088	0.130
7 RND	0.356	0.641	0.548	0.356	0.122	0.091	0.081	0.122
Lag days	SE avg	SP avg	ACC avg	PRE avg	SE sd	SP sd	ACC sd	SP sd
14	0.610	0.853	0.758	0.735	0.145	0.119	0.079	0.174
6	0.644	0.862	0.780	0.737	0.140	0.092	0.064	0.136
2	0.647	0.809	0.745	0.674	0.151	0.103	0.064	0.140

14 RND	0.373	0.635	0.547	0.373	0.127	0.095	0.089	0.127
6 RND	0.309	0.636	0.503	0.361	0.156	0.089	0.107	0.113
2 RND	0.353	0.611	0.525	0.353	0.141	0.102	0.092	0.141
Escalation date	SE avg	SP avg	ACC avg	PRE avg	SE sd	SP sd	ACC sd	SP sd
25 Mar	0.644	0.862	0.780	0.737	0.140	0.092	0.064	0.136
15 Apr	0.681	0.809	0.753	0.713	0.120	0.097	0.087	0.120
25 Mar RND	0.309	0.636	0.503	0.361	0.156	0.089	0.107	0.113
15 Apr RND	0.415	0.617	0.545	0.415	0.141	0.118	0.066	0.148
